# Honeycomb sterna: an unusual case of a developmental abnormality in the sternum

**DOI:** 10.1007/s00276-019-02350-4

**Published:** 2019-10-31

**Authors:** Cara Stella Hirst, S. White, T. Siek, A. Gasparik

**Affiliations:** 1 Anthropology Department, University College London, London, UK; 2 University College London, Institute of Archaeology, London, UK

**Keywords:** Sternum, Developmental abnormality, Variation, Unfused, Sternebrae, Accessory ossification centres

## Abstract

This report details an unusual case of a human sternal developmental abnormality of an anatomical specimen part of the skeletal collection curated by University College London, Anthropology Department skeletal collection. This rarely reported developmental abnormality is caused by the non-fusion of lateral ossification centres in the sternebrae, resulting in the mesosternum having a honeycomb-like appearance. Sternal defects are typically underreported in the clinical literature as many cases being asymptomatic that they are typically diagnosed incidentally, as such there is a dearth in our current understanding of the development and anatomical variants of the sternum. Although in recent years, large-scale CT studies have investigated the prevalence of sternal developmental abnormalities, these studies have not reported sternal defects similar to the individual presented in this report. While most sternal defects are clinically uneventful, the lack of awareness of these variants can result in misinterpretation of radiological and pathological findings as such an understanding of anatomical variants even when asymptomatic is vital.

## Introduction

**Fig. 1:**
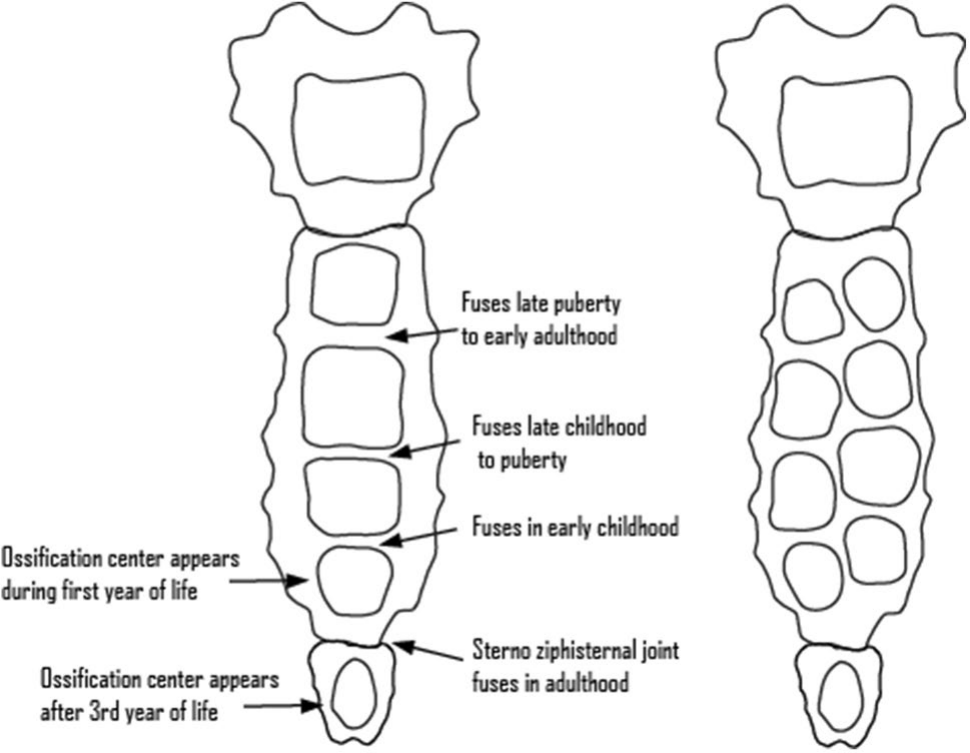
Diagram illustrating ossification centres of the sternum. (Left) Sternum with single ossification centres in the mesosternum and age ranges at which the sternebrae of the mesosternum fuse together. (Right) Segmented ossification centres in the mesosternum, which typically fuse together to form individual sternebrae at around 4 years of age

The adult sternum is comprised of three sections connected by secondary cartilaginous joints: the *manubrium* (the first sternebrae) located superiorly, the *mesosternum* (or body, comprised of the second to fifth sternebrae) in the centre, and the *xiphoid process* (the sixth sternebrae) inferior to these other elements [7–9, 14, 17]. Development of the sternum begins during the sixth week of intrauterine life, originating from a pair of longitudinal mesenchymal bands on either side of the anterior chest wall, which then migrate medially to form the cartilaginous sternum [9, 14]. Fusion of these two halves—known as the sternal bars—progresses from the cranial end to the caudal end of the sternum in a process which normally completes by the tenth week [2, 5, 10]. The cartilaginous sternum then ossifies from several ossification centres that appear in craniocaudal sequence around the fifth-to-sixth gestational weeks [14]. There are usually six ossification centres present in the sternum: one for the manubrium, four in the mesosternum, and one for the xiphoid process, though these numbers do vary somewhat (Fig. 1) [7, 17].

The mesosternum sternebrae are typically comprised of a single midline ossification centre. However, depending on the sternebra, it is not uncommon for more than one point of ossification to be present, an occurrence described as bifurcated ossification centres [7, 17]. The bifurcated centres in a single sternebra are usually arranged evenly and bilaterally across the midline, though they may also be somewhat displaced superiorly or inferiorly relative to one another [17]. When bifurcated (or multiple) ossification centres are present, the sternebrae initially exhibit osseous discontinuity, altered morphology of the adjacent margins, and/or asymmetrical forms. These ossification patterns are visible from the last month in utero until around 4 years of age when the separate sections of the sternum start to fuse [7]. Normally, these segments fuse during growth and it is often not possible to identify ossification patterns in adult individuals [1, 7].

Using radiography, Ashley [1] defined four patterns of sternal ossification, as follows:

Type I: From cranial to caudal orientation, the first three sternebrae of the mesosternum are usually formed of a single midline ossification centre. Vertical doubling of ossification centres may occur within these sternebrae, but they are still oriented along the midline. The fourth sternebra of the mesosternum may have a single or double ossification centre or may be absent entirely.

Type II: The first sternebra of the mesosternum alone or the first two sternebrae possess a single, midline ossification centre, while the second (some cases), third (all cases), and fourth (some cases) sternebrae may show double ossification centres. These doubled centres may be evenly oriented across the midline or displaced cranio-caudally.

Type III: The ossification centres in the first three sternebrae of the mesosternum are doubled (again, either evenly or displaced cranio-caudally) and the fourth sternebra may have either a single, double, or absent ossification centre.

Type IV: The first sternebra alone or first two mesosternal sternebrae have double ossification centres, while the third sternebra is a single centre and the fourth is single or absent.

Both abnormal development and incomplete fusion of the sternum are a rare finding in archaeology and anthropology, despite the variation in sternal morphology being well documented [5]. While fusion of the sternebrae is typically complete by 25 years of age [6], non-fusion of the sternebrae of the mesosternum has been reported among older individuals—though there have been no previously reported cases of non-fusion of multiple bifurcated sternal ossification centres as presented in this study (but see Knox [8]).

## Materials and methods

The sternum of an adult human from the Biological Anthropology collection housed in the University College London (UCL) Department of Anthropology was observed. The provenance of this sternum is unknown, although it is considered to have been part of a medical or anatomical collection. The primary evaluative framework for assessing the morphology of the sternum was macroscopic analysis. From these observations, the pattern of sternal fusion was classified as one of the four types using the criteria described by Ashley [1].

## Results

**Fig. 2:**
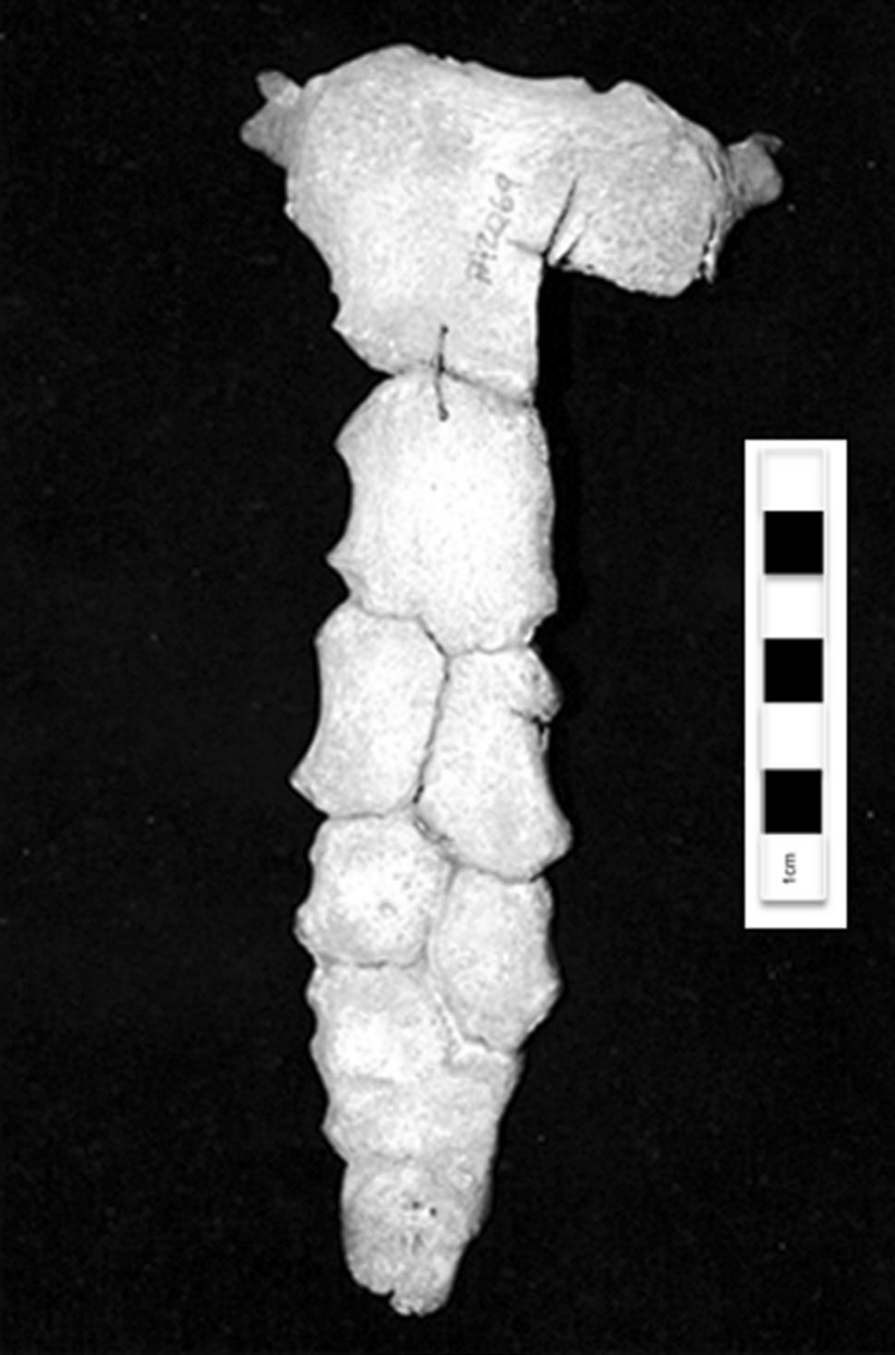
Human sternum with unfused accessory ossification centres in the mesosternum. Photograph of the anterior view of an adult human sternum including the manubrium, mesosternum, and ossified xiphoid process as well as ossified costal cartilage on the superior lateral aspects of the manubrium

The sternum, illustrated in Fig. 2, consisted of a manubrium and mesosternum made up of six segments showing varying degrees of fusion, along with the ossified xiphoid process and costal cartilage. While the manubrium and the first sternebra of the mesosternum were single fused sternebrae, the third and fourth were comprised of two unfused ossification centres, and the fifth sternebra appears to have originally had three ossification centres which had almost completely fused into a single section. This pattern of ossification is consistent with Type II based on the categories described by Ashley [1]. The sternal segments were vaguely hexagonal in morphology, but were longer along the superior–inferior axis and less regular in shape and size. The degree of fusion increased caudocranially, as is typical for *Homo sapiens* [6].

## Discussion

**Fig. 3:**
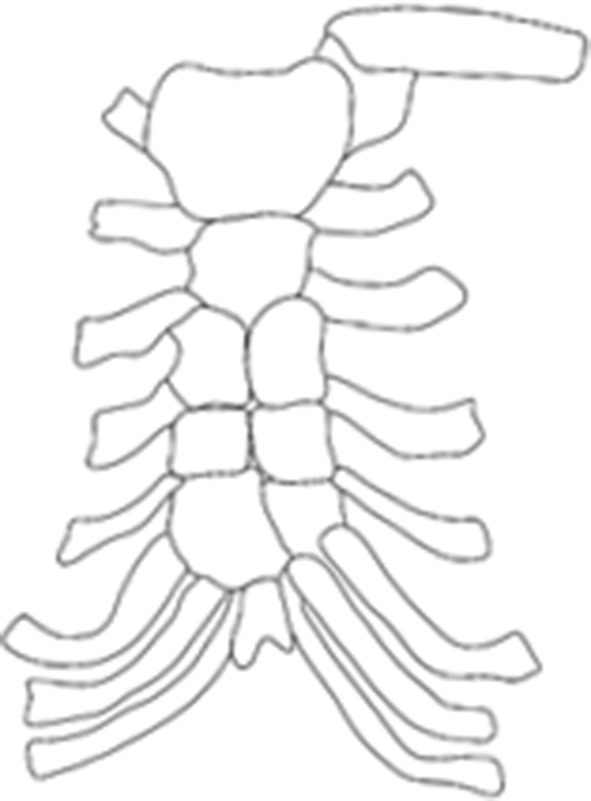
Sternum of an adult male human, illustration adapted from line drawing in Knox ([8]:293)

Sternal abnormalities are underdiagnosed in clinical settings, with clinically obvious cases typically diagnosed at or shortly after birth, and asymptomatic sternal abnormalities more often discovered incidentally later in life [14]. As a result, the majority of our understanding regarding the frequency and variation in sternal abnormalities comes from large-scale radiographic and Computed-Tomography (CT) imaging studies [17, 18]. These reviews have detailed numerous morphological variations and developmental abnormalities affecting the bones of the sternum [5, 18]. Yekeler et al. [18] reviewed the morphology and frequency of sternal variation and abnormalities in 1000 patients (582 men and 418 women; 20–92 years with a mean age of 54 years) who underwent thoracic multidetector CT examinations. Their results revealed the frequency of a number of previously documented sternal abnormalities such as sternal clefts, fissures, tubercles and fusion patterns, as well as several more unique cases such as a trifurcate xiphoid process, and an individual with three foramina in their xiphoid [18]. However, none of these previous, extensive, clinical reviews have identified individuals with developmental abnormalities similar to those reported here. The closest example that could be found in the literature was briefly described by Knox [8] (see Fig. 3) while commenting on the apparent frequency in which this developmental abnormality is found in specimens of *Pongo* [12, 15]. It consists of the sternum of an adult male human, with the second, third, and fourth mesosternal sternebrae each being separated into two segments.

CT examination of developing sterna in clinical contexts has found that accessory ossification centres are frequent in the manubrium and mesosternum [1, 7]. A review of 49 developing sternum reported bifurcated or accessory ossification centres among 20% of the individuals [10]. These accessory ossification centres form as a result of endochondral ossification and appear most frequently in the mesosternum [1]. While accessory ossification centres are typically symmetrical, they are known to develop in an irregular manner in instances of asymmetry in the costal joints [1]. As noted above, a review of ossification patterns reported four types of ossification. The most common form—which accounted for 60–67% of reported cases—involves one ossification centre in the manubrium, one in the first sternebrae, and two in the other sternebrae (Type II) [1]. Osseous fusion of the accessory ossification centres occurs prior to ossification of the mesosternebrae and is considered to be complete after 13 years of age; however, evidence of these ossification centres may remain evident in radiographic imaging [10].

Developmental abnormalities in the sternum resulting in non-fusion are common in the published literature and both sternal foramina and sternal clefts have been discussed extensively [13, 16, 18]. Sternal foramina are a relatively common developmental abnormality resulting from the incomplete fusion of a pair of sternebrae and have been reported to have a frequency of between 4.3 and 6.7% [4, 13, 16, 18]. As previously stated the sternal bones ossify from cartilaginous precursors that develop in craniocaudal succession from the fifth month in utero until shortly after birth [1, 3, 7]. It is a failure in this developmental process that results in various sternal abnormalities, such as sternal fissures and foramina [3].

While additional ossification centres in the mesosternum are a relatively common occurrence among juveniles and non-fusion of the sternebrae has been reported, to date, there have been no reported cases of non-fusion of these additional segments after 5 years of age. This report, therefore, details a previously undescribed developmental abnormality. Clinically, knowledge of the development, maturation, variation, and anomalies of the sternum is important in cases of potential chest and sternum injuries and surgical procedures [2, 11].

## Conclusion

This report details a human with a developmental sternal abnormality from the skeletal collection at University College London, Anthropology Department. The individual presented with a honeycomb-like appearance due to the non-fusion of the sternal ossification centres. This developmental abnormality has not been reported in the anthropological literature or in the extensive clinical reviews of sternal pathology and morphology. Due to lack of previous published cases of similar sternal abnormalities in previous clinical literature, it is considered unlikely that the defect identified in this report would present with clinically eventful symptoms; however, the lack of awareness of these variants can result in misinterpretation of radiological and pathological findings, and in rare instances fatality during surgical procedures and biopsies [16]. The sternum is frequently used as surgical entrance, particularly during cardiac surgery, as such increased awareness of structural and developmental variations is important. Due to the lack of contextual data, it is difficult to discuss the prevalence of this sternal abnormality or to draw specific conclusions. It may be possible that this honeycomb-like appearance may be more prevalent among other hominoid groups, but this can only be confirmed by re-examining anthropological and natural history collections.
